# Electrical Neurostimulation Promotes Brown Adipose Tissue Thermogenesis

**DOI:** 10.3389/fendo.2020.567545

**Published:** 2020-09-30

**Authors:** Zhuang Li, Wouter J. de Jonge, Yanan Wang, Patrick C. N. Rensen, Sander Kooijman

**Affiliations:** ^1^Department of Medicine, Division of Endocrinology, Leiden University Medical Center, Leiden, Netherlands; ^2^Einthoven Laboratory for Experimental Vascular Medicine, Leiden University Medical Center, Leiden, Netherlands; ^3^Tytgat Institute for Liver and Intestinal Research, Amsterdam UMC, Location AMC, Amsterdam, Netherlands; ^4^Department of Gastroenterology and Hepatology, Amsterdam UMC, Location AMC, Amsterdam, Netherlands; ^5^Department of Endocrinology, First Affiliated Hospital of Xi’an Jiaotong University, Xi’an Jiaotong University, Xi’an, China

**Keywords:** brown adipose tissue, neurostimulation, thermogenesis, beta-3 adrenergic receptor signaling, brown adipose tissue temperature

## Abstract

**Background:**

Brown adipose tissue (BAT) is present in humans and rodents, and contributes to energy expenditure by converting energy stored in lipids and glucose into heat. Beta adrenergic receptor (β-AR) agonists have been proposed as pharmacological tools to activate BAT, but they lack selectivity for this tissue. This study aimed to investigate the possibility to apply electrical neurostimulation as a novel approach to activate BAT by promoting the sympathetic outflow towards BAT.

**Methods:**

Male C57BL/6J mice were treated with either unilateral electrical neurostimulation of interscapular BAT or with the β3-AR agonist CL316,243. Thermogenesis, nutrient uptake by BAT and downstream signaling of adrenergic receptors in BAT were examined.

**Results:**

Electrical neurostimulation and β3-AR agonism acutely increased heat production by BAT, as evidenced by an increase in local temperature in BAT, without influencing the core body temperature. Both treatments acutely increased tyrosine hydroxylase content in the nerve terminals thereby confirming enhanced sympathetic activity. In addition, we identified increased phosphorylation of hormone-sensitive lipase coinciding with reduced intracellular lipids in BAT, without affecting acute nutrient uptake from plasma. The increased BAT temperature as induced by electrical neurostimulation was reversed by β3-AR antagonism.

**Conclusion:**

Electrical neurostimulation acutely promotes thermogenesis in BAT as dependent on β3-AR signaling. We anticipate that electrical neurostimulation may be further developed as a novel strategy to activate BAT and thereby combat (cardio)metabolic diseases.

## Introduction

Brown adipose tissue (BAT) is a metabolically active tissue with a crucial role in thermogenesis in small rodents and infants, but is yet also present and active in human adults (1, 2). Human studies identified a negative correlation between BAT activity and BMI/fasting glucose, suggesting that BAT is an important tissue for glucose homeostasis and a potential therapeutic target to combat (cardio)metabolic diseases (3, 4).

The physiological activator of BAT is cold exposure (5, 6). Mechanistically, upon cold exposure, sympathetic outflow to BAT increases local norepinephrine production and release, which activates β-adrenergic receptors (β-AR) on the brown adipocyte to promote an intracellular signaling cascade. Via the production of cyclic AMP (cAMP) and activation of protein kinase A (PKA), β-AR signaling stimulates lipolysis as well as the transcription of genes involved in thermogenesis (7). Fatty acids (FAs) that are released upon intracellular lipolysis allosterically activate uncoupling protein-1 (UCP1) and serve as fuel for non-shivering thermogenesis (8). To replenish lipid stores, brown adipocytes take up large amounts of triglyceride (TG)-derived FAs and glucose (9). In addition, accelerated TG-rich lipoprotein turnover stimulates reverse cholesterol transport (10). The combined effect is attenuated dyslipidemia and atherosclerosis development.

The β3-adrenergic receptor (β3-AR) is the dominant adrenergic receptor in murine BAT, while the β2-adrenergic receptor is most likely responsible for promoting thermogenesis in human BAT (11). Independent of the ongoing debate about the relative contributions of the various β-ARs in human BAT function, targeting any of the β-ARs with the goal to activate BAT will be challenging given their critical involvement in the cardiovascular and pulmonary systems. Thus, a different approach to selectively activate BAT to combat (cardio)metabolic diseases is highly warranted.

As an alternative for the use of sympathomimetics, one might think of promoting endogenous sympathetic outflow to BAT. Previous studies demonstrated that electrical stimulation of specific hypothalamic nuclei can promote sympathetic outflow and as a results BAT thermogenesis (12, 13). In addition, electrical field stimulation of the dorsal surface of interscapular BAT was shown to cause an acute increase in BAT temperature (14). Moreover, others have used local optogenetics to selectively promote activity of the tyrosine hydroxylase (TH)-expressing neurons innervating BAT, which suggested that stimulation of the sympathetic nerves is sufficient to elicit thermogenesis in BAT (15). However, the main disadvantage of optogenetics is the hurdle to take such an approach to the clinic.

In the current study, we have taken a state of art approach by applying electrical neurostimulation to specifically promote outflow of the postganglionic sympathetic nerves that innervate BAT in mice. This is the first step toward the use of implantable devices that can very specifically promote BAT thermogenesis.

## Materials and Methods

### Animals

Male C57Bl/6J mice at the age of 12 to 16 weeks were housed under standard conditions with a 12-h light/dark cycle (lights on 07.00h; lights off 19.00h) with *ad libitum* access to regular chow diet and water. At the beginning of the experiments, mice were randomly divided into the respective groups. Animal experiments were performed under approval by the Ethics Committee on Animal Care and Experimentation of the Leiden University Medical Center and following the regulations of the Dutch law on animal welfare.

### Temperature Recording

Mice were anesthetized by inhalation of isoflurane (4%, v/v air) for 3 min, and their backs were shaved. Because anesthesia affects thermoregulation, the mice were placed on a heating plate (HP-4M Small Animal Heating Plate, Physitemp) that was connected to a temperature controller (TCAT -2LV; Physitemp), while being kept under anesthesia by isoflurane (<2.5%, v/v air) during the whole subsequent intervention period. A rectal temperature probe (RET-3 Rectal Probe, Physitemp) connected to the temperature controller was inserted into the rectum of mice to measure their core body temperature. Their core body temperature was set to be stable at approx. 36.6°C by automatically switching the heating plate on and off. Approached from the back, both pads of interscapular BAT (left and right) were exposed by a midline incision in the skin and white fat along the upper dorsal surface. Flexible probes (IT-18 Flexible Implantable Microprobe, Physitemp) were plugged into both the left and right BAT pad to monitor the local temperature in BAT. The core body temperature and temperature in BAT were recorded per second during the whole intervention by a sensitive temperature data acquisition system (THERMES-USB; Physitemp) connecting to a laptop using Dasylab software (Version 12.0).

### Pharmacological Stimulation of BAT

10 min after starting temperature monitoring, mice received either the β3-adrenergic receptor (β3-AR) agonist CL316,243 (CL; Tocris Bioscience Bristol, United Kingdom; 20 μg/mouse) or vehicle (phosphate-buffered saline; PBS) by subcutaneous injection (100 µl/mouse).

### Electrical Neurostimulation of BAT

**Figure 1:**
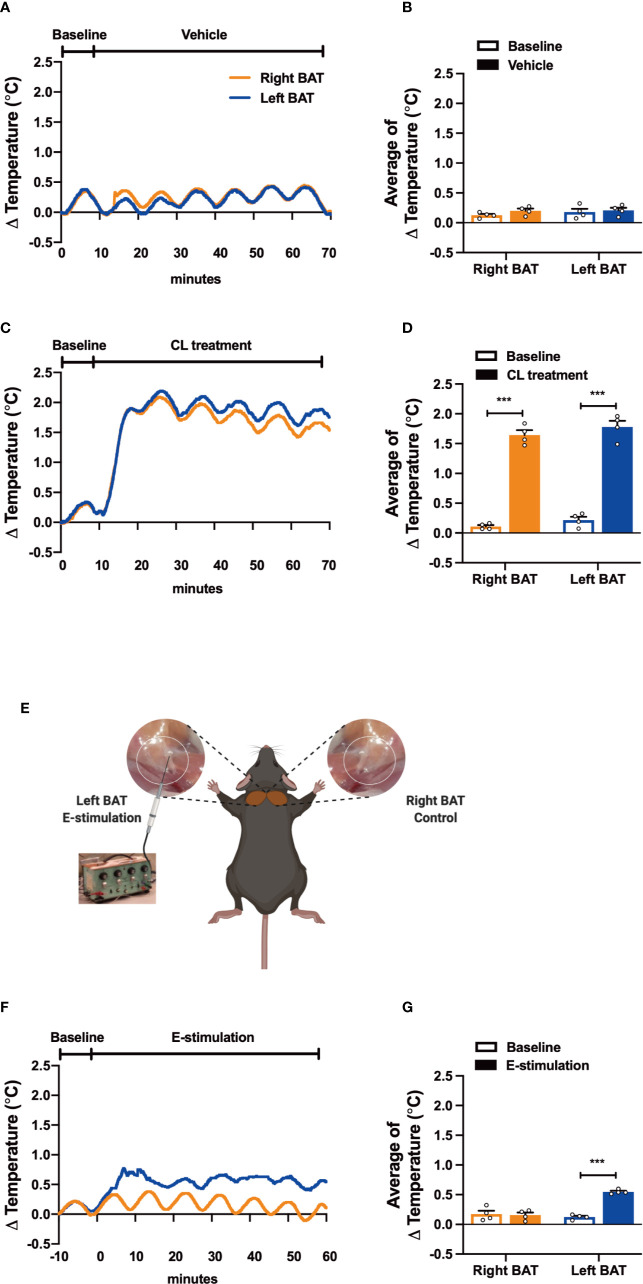
Electrical neurostimulation acutely increases the thermogenic activity of BAT. After recording local BAT temperature for 10 min (baseline), mice received vehicle **(A, B)**, CL316243 (CL treatment) **(C, D)**, or electrical sympathetic neural stimulation (E-stimulation) of the left BAT lobe with the right lobe as unstimulated control (schematically shown; **E**) for 60 min **(F, G)**, during which BAT temperature was still recorded. The temperature changes **(A, C, F)** and average temperature changes within the intervention periods **(B, D, G)** were calculated. Differences between groups were determined with a two-tailed Student unpaired t-test. Data are shown as average curves **(A, C, F)** or mean ± SEM **(B, D, G)** (n=4 mice per group). ***P<0.001.

For unilateral electrical neurostimulation of interscapular BAT, the sympathetic nerves of the left BAT pad were connected to an electrical stimulator by using a homemade hook electrode. 10 min after the start of temperature monitoring, mice received continuous sympathetic neural stimulation with 4 V, 2 ms pulses at 6 Hz for 60 min. The sympathetic nerves of the right BAT pad were just exposed by sham operation (control). The experimental procedure is depicted in **Figure 1E**. When indicated, mice received the β3-AR antagonist SR59230A (Sigma-Aldrich; 1 μM; 100 µl/mouse) by subcutaneous injection after 30 min of electrical stimulation.

### *In Vivo* Triglyceride and Glucose Clearance

TG-rich lipoprotein (TRL)-like particles (80 nm), radiolabeled with glycerol tri[^3^H]oleate (3.7 MBq) were prepared as described before (16), and stored at 4°C under argon until use at the second day after preparation. TRL-like particles were mixed 2-[1-^14^C]deoxy-D-glucose ([^14^C]DG) in a 4:1 ratio (^3^H:^14^C). 15 min before the end of the pharmacological or electrical intervention, mice were intravenously injected *via* the tail vein with the combination of TRL-like particles (1 mg TG) and deoxyglucose (200 µl/mouse). After 15 min, mice were killed by CO_2_ inhalation, perfused with ice-cold PBS, and both left and right interscapular BAT was collected and a piece of each BAT pad was dissolved in 500 µl of Solvable (Perkin Elmer) overnight at 56°C. The uptake of ^3^H and ^14^C activity by BAT was determined using scintillation counting (Ultima Gold XR, Perkin Elmer).

### BAT Histology

At the end of the pharmacological or electrical neurostimulation, both left and right interscapular BAT were harvested and another piece of each BAT pad was snap-frozen in liquid N_2_ and subsequently stored at −80°C until histological analysis. BAT cryostat sections (10 µm) were cut and stained with hematoxylin and eosin (H&E) using a standard protocol, and stained for tyrosine hydroxylase (TH, 1/2000; Ab112; Abcam) and phospho-hormone sensitive lipase (HSL, Ser563, 1/2000; #4139; Cell Signaling). The areas occupied by intracellular lipid vacuoles, TH and HSL (positive area per total area) were quantified using Image J software.

### Western Blotting

Pieces of BAT were lysed in RIPA buffer (25 mM Tris-HCl pH 7.6, 150 mM NaCl, 1% NP-40, 1% sodium deoxycholate and 1% SDS; Thermo Fisher Scientific) supplemented with Complete Protease and phosSTOP phosphatase inhibitors (Roche Diagnostics) with the Qiagen TissueLyser II (Qiagen). Protein concentrations were determined using a bicinchoninic acid (BCA) assay (Thermo Fisher Scientific). Western blots were carried out on the Wes system (ProteinSimple) following the manufacturer’s instructions using the primary antibody of tyrosine hydroxylase (TH, 1/250; Ab112; Abcam) and phospho-hormone-sensitive lipase (HSL, Ser563, 1/500; #4139; Cell Signaling). Protein expression levels were normalized to GAPDH (1/1000; #25778; Santa Cruz) housekeeping protein expression. Western blot quantifications were done with Image J software.

### Statistical Analysis

Differences between the groups were determined with a two-tailed Student unpaired t-test. Data on temperature changes are shown as average curves, and average temperature changes per period are calculated and shown as mean ± SEM (n=4 mice per group). Statistical analysis was performed using GraphPad Prism 8. P-values <0.05 were considered significant.

## Results

### Electrical Neurostimulation Acutely Increases the Thermogenic Activity of BAT

To test our experimental set-up, we first evaluated the effect of pharmacological activation on the thermogenic capacity of intrascapular BAT. Thereto, mice were sedated, connected to the temperature maintenance recording system and received a subcutaneous injection with either β3-AR agonist CL316243 (CL treatment) or PBS (vehicle). While the temperature of both BAT pads was not affected by vehicle treatment (**Figures 1A, B**), CL treatment acutely increased the temperature in the tissue (right BAT: +1.53°C, left BAT: +1.56°C, **Figures 1C, D**). Core body temperature was not affected by the treatment, which was expected as the mice were sedated and body temperature was kept stable using an automated heating plate combined with a rectal temperature probe (**Figures S1A, B**).

Next, to assess the effect of electrical neurostimulation (E-stimulation) on the thermogenic activity of BAT, a hook electrode was unilaterally connected to the sympathetic nerves innervating the left interscapular BAT as schematically shown in **Figure 1E**. During E-stimulation, the temperature of the stimulated left BAT pad was consistently increased by on average +0.42°C when compared to baseline recordings. In contrast, the temperature of the unstimulated right BAT pad was not increased during this period (**Figures 1F, G**). Similar to the pharmacological stimulation of BAT, E-stimulation did not influence core body temperature (**Figure S1C**).

### Electrical Neurostimulation Reduces Intracellular Lipid Droplets in BAT

**Figure 2:**
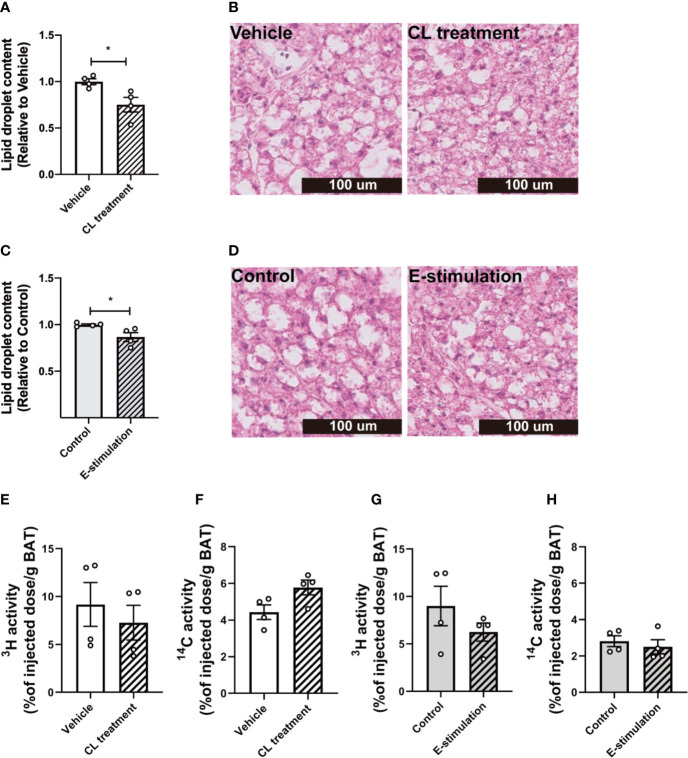
Electrical neurostimulation reduces intracellular lipid droplets in BAT. At 60 min of intervention with vehicle or CL316243 **(A, B)**, or electrical neural sympathetic stimulation (E-stimulation) of the left BAT lobe **(C, D)**, BAT was collected and sectioned for HE staining. The lipid content within BAT was quantified **(A, C)** with representative pictures shown **(B, D)**. Lipid and glucose uptake by BAT was assessed by injection of glycerol tri[^3^H]oleate-labeled triglyceride-rich lipoprotein-mimicking particles and [^14^C]deoxyglucose injection, 15 min before termination. The uptake of ^3^H and ^14^C-activity by BAT were assessed in mice receiving vehicle versus CL316243 **(E, F)** and E-stimulation of the left lobe **(G, H)**. Differences between groups were determined with a two-tailed Student unpaired t-test. Data are shown as mean ± SEM (n=4 mice per group). *P<0.05.

After 1 h of pharmacological or electrical neurostimulation of BAT, the mice were injected with radiolabeled lipoprotein-like particles to determine TG-derived FA uptake by BAT and tissues were collected for further analysis. We observed a marked decrease of lipid droplet content in BAT of the mice receiving CL treatment as compared with the vehicle (−25%, **Figures 2A, B**). The BAT pad of mice that had received E-stimulation also showed a significant decrease in lipid droplet content (−13%, **Figures 2C, D**). Despite the decrease in lipid content, there was no effect on the uptake of [^3^H]oleate or [^14^C]deoxyglucose by BAT of the CL treated mice (**Figures 2E, F**), nor was there in the E-stimulated BAT depot (**Figures 2G, H**).

### Electrical Neurostimulation Acutely Increases Tyrosine Hydrolase and Hormone-Sensitive Lipase Phosphorylation in BAT

**Figure 3:**
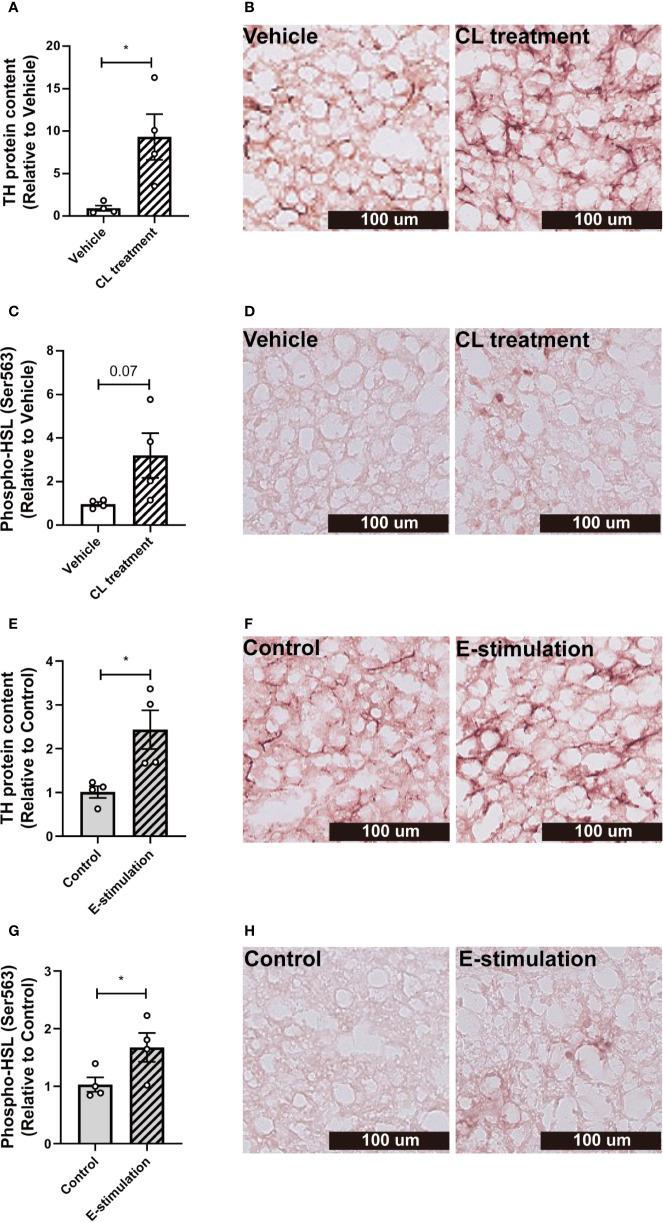
Electrical neurostimulation acutely increases tyrosine hydrolase and hormone-sensitive lipase phosphorylation in BAT. After intervention with vehicle or CL316243 **(A–D)**, or electrical neural sympathetic stimulation (E-stimulation) of the left BAT lobe **(E–H)**, BAT was collected and sectioned for immunohistochemical staining. The protein expression of tyrosine hydrolase (TH) **(A, B, E, F)** and phospho-hormone sensitive lipase (HSL) **(C, D, G, H)** were quantified **(A, C, E, G)** with representative pictures shown **(B, D, F, H)**. Differences between the groups were determined with a two-tailed Student unpaired t-test. Data are shown as mean ± SEM (n=4 mice per group). *P<0.05.

To investigate whether norepinephrine production and downstream signaling of adrenergic receptors were promoted by E-stimulation, we next quantified protein levels of tyrosine hydroxylase (TH), the rate-limiting enzyme in norepinephrine production and phosphorylation of hormone-sensitive lipase (HSL) Ser563, essential for the intracellular lipolysis of TG. CL treatment increased the TH content (Histology: 9 fold, **Figures 3A, B**; Western blot: 3.6 fold, **Figures S3A, B**), possibly as part of a positive feedback loop and explained by rapid axoplasmic transport from the cell bodies to the terminals. This effect was accompanied by a nonsignificant increase in phosphorylated HSL (**Figures 3C, D**, **S3D, E**). E-stimulation resulted in an increased TH level (Histology: 2.4 fold, **Figures 3E, F**; Western blot: 3.6 fold, **Figures S3A, C**) and a significant increase in phosphorylated HSL (Histology: 1.7 fold, **Figures 3G, H**; Western blot: 3.1 fold, **Figures S3D, F**).

### The Effect of Electrical Neurostimulation on the Thermogenic Activity of BAT is Dependent on β3-Adrenergic Receptor Signaling

**Figure 4:**
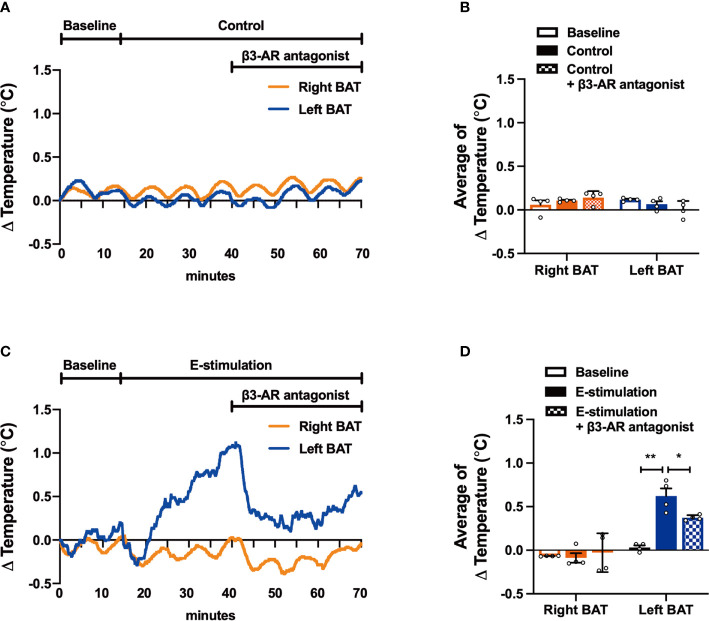
The effect of electrical neurostimulation on the thermogenic activity of BAT is dependent on β3-adrenergic receptor signaling. After recording local BAT temperature for 10 min (baseline), mice were sham-operated as control **(A, B)** or received electrical neural sympathetic stimulation (E-stimulation) of specifically the left BAT lobe for 60 min **(C, D)**, during which BAT temperature was still recorded. After 30 min of intervention, all mice in addition received a β3-adrenergic (β3-AR) antagonist by subcutaneous injection. The temperature changes **(A, C)** and the average temperature changes in different intervention periods **(B, D)** were calculated in control mice **(A, B)** and in mice receiving E-stimulation **(C, D)**. Differences between the groups were determined with a two-tailed Student unpaired t-test. Data are shown as mean ± SEM (n=4 mice per group). *P<0.05, **P<0.01.

To confirm that electrical neurostimulation indeed results in enhanced sympathetic outflow and thereby promotes thermogenesis, we repeated E-stimulation while concomitantly administering a specific β3-AR antagonist. The β3-AR antagonist SR59230A had no effect on BAT temperature in sham-operated mice (**Figures 4A, B**), but acutely reduced the increased temperature in the E-stimulated BAT pad (**Figures 4C, D**). There was no effect on core body temperature during E-stimulation or treatment with the β3-AR antagonist (**Figures S2A, B**).

## Discussion

Here, we have described a novel method of applying electrical neurostimulation to selectively promote sympathetic outflow to BAT in mice. In this proof of concept study, by using C57BL/6J mice we demonstrated that electrical neurostimulation of BAT promotes thermogenesis dependent on β3-AR signaling. Although we are not the first to target BAT by electrical stimulation, previous studies used techniques that were nonspecific (i.e. electrical field stimulation of dorsal surface)) (14), very difficult to translate to the clinic (i.e. with the use of optogenetics) (15) or a combination of these two limitations (i.e. electrical stimulation of hypothalamic nuclei) (12, 13). The method described in our manuscript involves electrodes that are directly positioned around the sympathetic nerves innervating BAT; this is the first step towards the use of implantable devices to selectively promote thermogenesis in BAT.

In contrast to other organs such as white adipose tissue and liver, BAT is densely innervated. Almost every single brown adipocyte is in close proximity to a sympathetic nerve ending, as also reflected by the abundant TH staining shown in the present study. This not only suggests that BAT is under stringent control of the nervous system, but is also consistent with the critical role of BAT in the acute response and tolerance to cold. Indeed, in line with the previous studies showing that the activation of BAT acutely increases intracellular lipolysis to release FA that serve as fuel for thermogenesis (8), in the current study we demonstrated that electrical neurostimulation of BAT acutely increases the thermogenic capacity of BAT. Although 1 h of electrical stimulation was seemingly insufficient to already promote uptake of lipids and glucose from the circulation, given that both acute CL injection and electrical neurostimulation did lead to decreased lipid content in BAT, and chronic CL treatment does attenuate diet-induced adiposity, hyperlipidemia and atherosclerosis (10, 16), we anticipate that prolonged neurostimulation would be a feasible alternative to activate BAT and prevent (cardio)metabolic diseases as shown for the use of sympathomimetics (9). More studies are needed to further explore the potential application of prolonged electrical neurostimulation of BAT preferably in free-living animals by using cuff electrodes connected to a swivel.

Electrical stimulation of peripheral nerves is already applied in a variety of conditions in humans (17, 18). Evidently, the approach used in the current study, which involves surgery to expose the nerves innervating BAT and wired connections to a pulse generator, is not suitable for clinical application yet. In addition, important questions related to the similarities between BAT physiology of humans (19) and rodents (20), as well as the effects of prolonged and/or repeated neurostimulation have to be addressed first. Interestingly, a human study involving vagus nerve stimulation, used to treat refractory epilepsy, also demonstrated increased energy expenditure and weight loss in association with increased BAT activity (21), highlighting the potential of neurostimulation in the clinical treatment of cardiometabolic disorders.

In summary, we demonstrated that direct electrical stimulation of the sympathetic nerves innervating BAT potently induces heat production, which is dependent on β3-AR signaling. Future studies should show whether prolonged and/or repeated neurostimulation of BAT, preferentially using implantable devices, can protect from (cardio)metabolic diseases.

## Data Availability Statement

The raw data supporting the conclusions of this article will be made available by the authors, without undue reservation.

## Ethics Statement

The animal study was reviewed and approved by Ethics Committee on Animal Care and Experimentation of the Leiden University Medical Center.

## Author Contributions

ZL: study concept and design, acquisition of data, analysis and interpretation of data, draft manuscript. WdJ, YW: study concept and design, interpretation of data. PR, SK: study concept and design, interpretation of data, obtained funding, study supervision. All authors contributed to the article and approved the submitted version.

## Funding

This work was supported by an Innovation Grant of the Dutch Heart Foundation (2015T092 to PR and SK).

## Conflict of Interest

The authors declare that the research was conducted in the absence of any commercial or financial relationships that could be construed as a potential conflict of interest.
