# Supplementary material for: Electrical Neurostimulation Promotes Brown Adipose Tissue Thermogenesis
Source: Front Endocrinol (Lausanne). 2020 Sep 30;11:567545. doi: 10.3389/fendo.2020.567545 (PMC7555256; doi:10.3389/fendo.2020.567545)
Supplement: Supplementary file 1 [file DataSheet_1.pdf]

Supplementary Figures

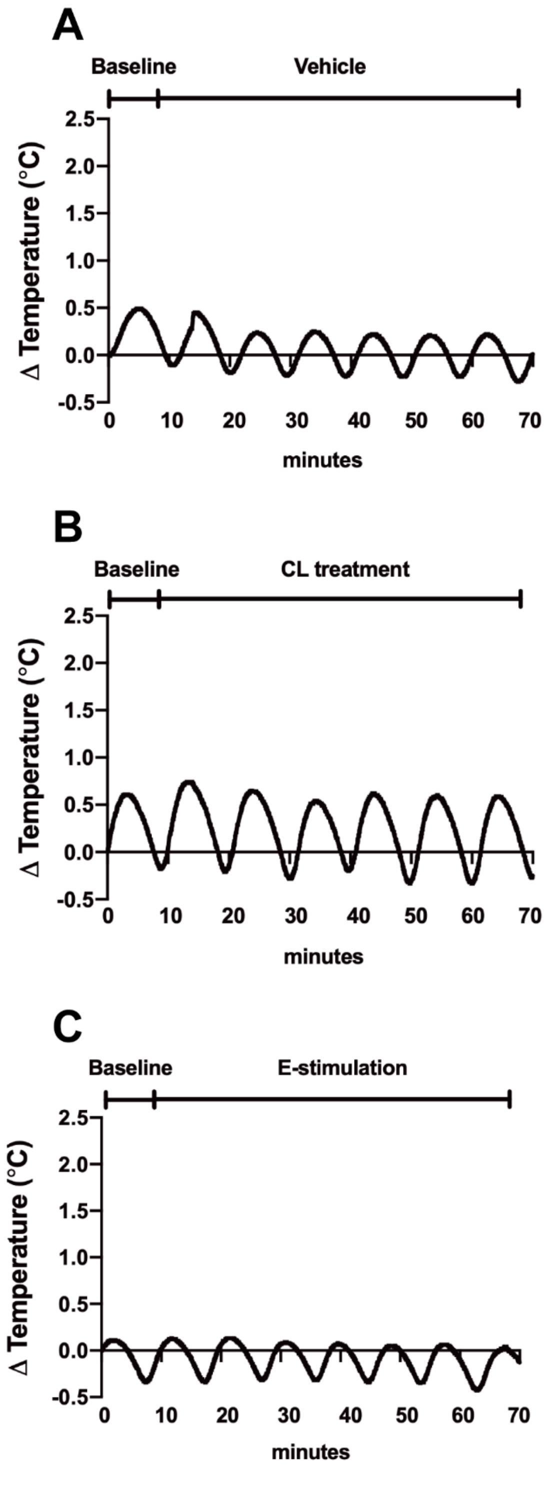

Figure S1. Electrical neurostimulation does not influence the core body temperature. After recording core body temperature for 10 min (baseline), mice received vehicle (A), CL316243 (CL treatment) (B), or electrical neurostimulation (E-stimulation) of the left BAT lobe for 60 minutes (C), during which core body temperature was still recorded. The temperature changes were calculated as average curves (n=4 mice per group).

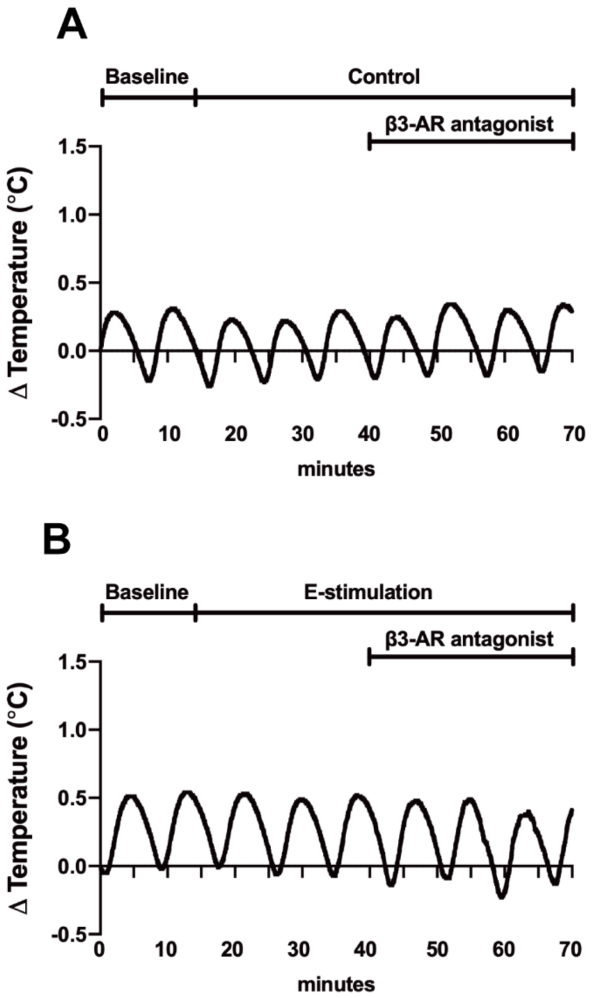

Figure S2. β3-adrenergic antagonism without and with electrical neurostimulation does not influence the core body temperature. After recording core body temperature for 10 min, mice were sham-operated (control, A) or received electrical neurostimulation (E-stimulation) of the left BAT lobe for 60 minutes (B), during which core body temperature was still recorded. After 30 min of intervention, all mice in addition received a β3-adrenergic (β3-AR) antagonist by subcutaneous injection. The temperature changes were calculated as average curves (n=4 mice per group).

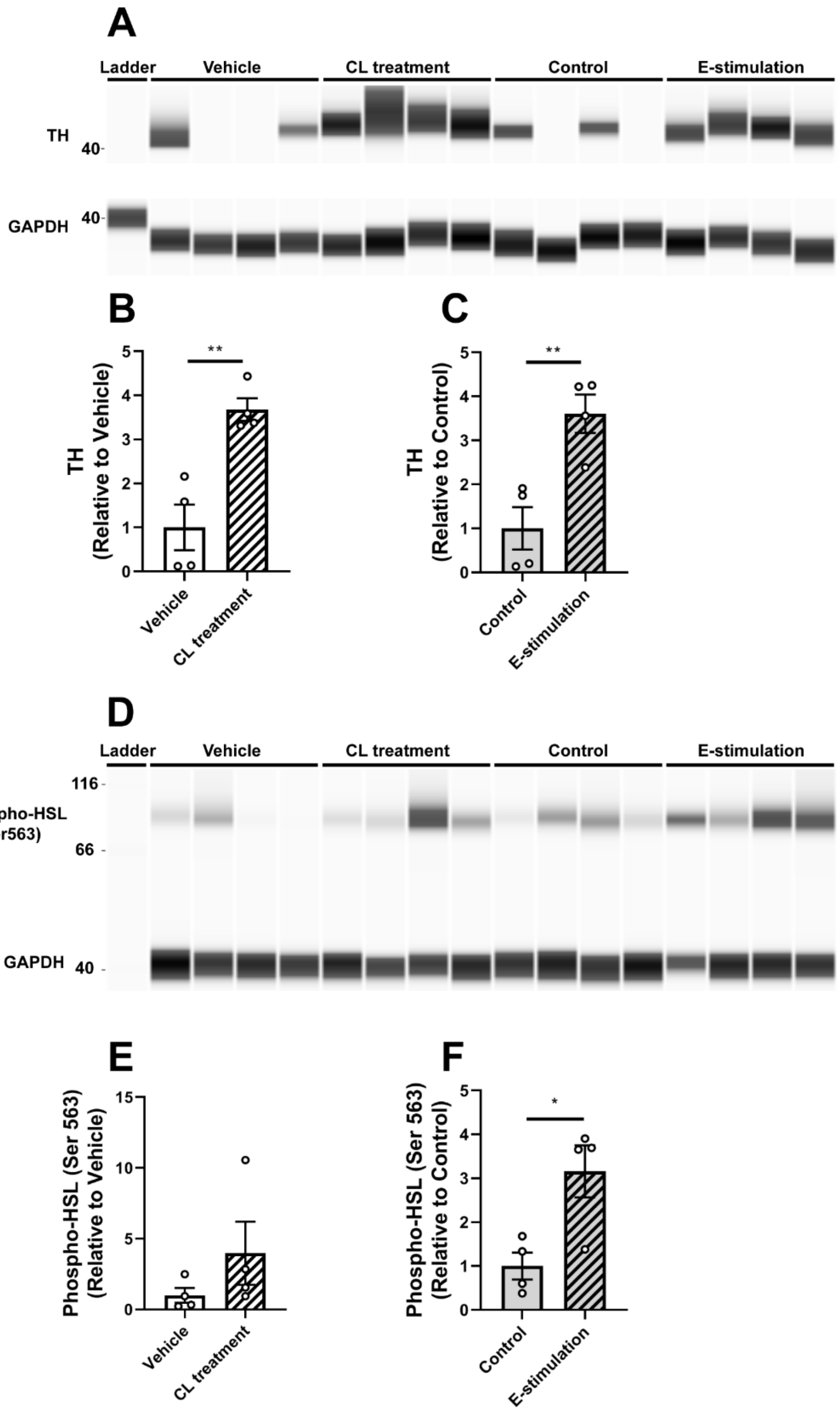

Figure S3. Electrical neurostimulation acutely increases tyrosine hydroxylase and phosphorylated hormone-sensitive lipase in BAT. After intervention with vehicle or CL316243, or electrical neural sympathetic stimulation (E-stimulation) of the left BAT lobe, BAT was collected and lysed for western blot. The protein level of tyrosine hydroxylase (TH) (A) and phospho-hormone-sensitive lipase (HSL) (D) were quantified (B, C, E, F). Differences between the groups were determined with a two-tailed Student unpaired t-test. Data are shown as mean ± SEM (n=4 mice per group). *P<0.05, **P<0.01.
